# Transcriptome Analysis Identifies Doublesex and Mab-3 Related Transcription Factor (DMRT3) in Nasal Polyp Epithelial Cells of Patients Suffering from Non-Steroidal Anti-Inflammatory Drug-Exacerbated Respiratory Disease (AERD)

**DOI:** 10.3390/biom11081092

**Published:** 2021-07-23

**Authors:** V.S. Priyadharshini, Marcos Alejandro Jiménez-Chobillon, Jos de Graaf, Raúl Porras Gutiérrez de Velasco, Christina Gratziou, Fernando Ramírez-Jiménez, Luis M. Teran

**Affiliations:** 1 Instituto Nacional de EnfermedadesRespiratorias Ismael Cosío Villegas, Calz. de Tlalpan 4502, Belisario Domínguez Secc 16, Mexico City 14080, Mexico; js.prriya@gmail.com (V.S.P.); aljicho@gmail.com (M.A.J.-C.); dr_frj@yahoo.com.mx (F.R.-J.); 2 Translational Oncology at Johannes Gutenberg-University Medical Center gGmbH, D-55131 Mainz, Germany; Jos.degraaf@tron-mainz.de; 3 School of Medicine, Universidad Nacional Autónoma de México, Av. Universidad 3000, Circuito Exterior S/N. Delegación Coyoacán, Mexico City 04510, Mexico; raul.porras@cannapeutas.mx; 4 Smoking Cessation Centre Pulmonary Department, Evgenidio Hospital, Athens University, 20 Papadiamantopoulou Street, 11528 Athens, Greece; chgratziou@yahoo.com

**Keywords:** Aspirin-exacerbated respiratory disease, DMRT3, epithelial cells, nasal airway, nasal polyps, transcriptome analysis

## Abstract

Background: Aspirin-exacerbated respiratory disease (AERD) is a syndrome characterised by chronic rhinosinusitis, nasal polyps, asthma and aspirin intolerance. An imbalance of eicosanoid metabolism with anover-production of cysteinyl leukotrienes (CysLTs) has been associated with AERD. However, the precise mechanisms underlying AERD are unknown. Objective: To establish the transcriptome of the nasal polyp airway epithelial cells derived from AERD patients to discover gene expression patterns in this disease. Methods: Nasal airway epithelial cells were isolated from 12 AERD polyps and 8 AERD non-polyp nasal mucosa samples as controls from the same subjects. Utilising the Illumina HiSeq 2500 platform, RNA samples were sequenced. Potential gene candidate DMRT3 was selected from the differentially-expressed genes for validation. Results: Comparative transcriptome profiling of nasal epithelial cells was accomplished in AERD. A total of 20 genes had twofold mean regulation expression differences or greater. In addition, 8 genes were upregulated, including doublesex and mab-3 related transcription factor 3 (DMRT3), and 12 genes were downregulated. Differentially regulated genes comprised roles in inflammation, defence and immunity. Metabolic process and embryonic development pathways were significantly enriched. Enzyme-linked immune sorbent assay (ELISA) results of DMRT3 in AERD patients were significantly upregulated compared to controls (*p* = 0.03). Immunohistochemistry (IHC) of AERD nasal polyps localised DMRT3 and was predominantly released in the airway epithelia. Conclusion: Findings suggest that DMRT3 could be potentially involved in nasal polyp development in AERD patients. Furthermore, several genes are downregulated, hinting at the dedifferentiation phenomenon in AERD polyps. However, further studies are imperative to confirm the exact mechanism of polyp formation in AERD patients.

## 1. Introduction

Aspirin-exacerbated respiratory disease (AERD) is a syndrome characterized by rhinosinusitis, nasal polyps, asthma and aspirin intolerance. AERD is also known as non-steroidal anti-inflammatory drug-exacerbated respiratory disease (N-ERD), aspirin-sensitive asthma or aspirin-intolerant asthma. Up to 73% of AERD patients develop atopy, although specific IgE antibodies to aspirin have not been identified. The pathogenic mechanisms associated with AERD include overproduction of cysteinyl leukotrienes (CysLTs), increased CysLTR1 expression in the airway mucosa and decreased lipoxin and PGE2 synthesis [1,2,3]. Anti-leukotriene therapy ameliorates asthma symptoms in aspirin-intolerant patients. However, nasal polyp progression remains a significant challenge in AERD management. Nasal polyposis is characterized by inflammatory pseudotumoural masses that most frequently start to grow from the ostiomeatal complex and the cells of the anterior ethmoidal sinus. They can affect the totality of the remaining sinus cavities, including the posterior ethmoidal cells and the maxillary, frontal or sphenoidal sinuses; they also can extend to the olfactory cleft, the sphenoethmoidal recess, and the nasal cavities [4]. Nasal polyposis occurs in up to 80 to 90% of AERD patients and tends to be more aggressive and difficult to treat medically, presenting with higher recurrence rates after surgery. A survey in 190 AERD patients suffering from NPs that analysed perceptions and quality of life showed that chronic nasal symptoms subsequently caused a decreased sense of smell in these patients. This study reported that NPs have the most significant impact on quality of life (40%, approximately), and patients who lost their smell (34%) reported that they missed the enjoyment of food [5]. On the other hand, the surgical removal of nasal polyps has been demonstrated to decrease both urinary LTE4 levels and asthma exacerbations [6,7,8].

The mucosal lining of the nasal polyps is a columnar glandular pseudostratified epithelium that plays a significant role in cytokine and inflammatory mediator release and has been implicated in nasal polyps in AERD. A study conducted by Picado et al. showed that COX-2 was downregulated in NP epithelial cells (ECs) derived from aspirin-sensitive patients and proposed that dysregulation of COX-2 could serve a crucial role in nasal polyps [9]. Subsequently, Kowalski et al. found NP ECs from AERD patients generated three-fold less prostaglandin E2 (PGE2) than aspirin-tolerant subjects. Prostaglandin E2 (PGE2) possesses bronchodilator properties; decreased production of this mediator could account for AERD development [10]. In 2007, the same group also investigated the ability of nasal polyp epithelial cells to produce higher levels of stem cell factor when compared with aspirin-tolerant patients; they proposed that increased expression and secretion of stem cell factor, a chemotactic growth and differentiation factor for mast cells, accounted for increased mast cell infiltration and activation [11] in AERD. In this study, we have investigated AERD polyps’ epithelial cell transcriptome.

## 2. Methods

### 2.1. Study Design

Subjects were divided into three groups. Group A subjects (*n* = 12) included AERD patients who underwent routine polypectomy for a therapeutic reason. Two types of samples were collected from these patients: nasal polyps which were removed during polypectomy (*n* = 12); and nasal tissue from the middle turbinate, which was collected from the non-polyp nasal mucosa (*n* = 8). These samples were used to isolate epithelial cells for RNA sequencing experiments. In addition, group B, which was composed of AERD patients (*n* = 12), and group C, which was composed of non-atopic, healthy controls (*n* = 8), were included for validation experiments: nasal lavages were performed in these two groups of patients for ELISA measurements.

### 2.2. Subjects

Thirty-two subjects participated in this study (Table 1). Their atopic status was investigated by skin-prick testing with different allergens. AERD was defined as the presence of asthma, nasal polyps or previous polyp surgery, and NSAID intolerance (a nasal challenge with lysin-aspirin or two severe reactions to NSAID previously). Asthma was established as typical persistent symptoms of shortness of breath, wheezing, chest tightness, and cough, plus >12% or 200 mL increase of forced expiratory volume in 1 s (FEV1) during post-bronchodilator spirometry (Master Screen, Jaegger, Hoöchberg Germany). Allergy sensitisation was evaluated with a skin prick test with a kit of 40 allergens (Alk-abello; Round Rock, TX, USA), and levels of total IgE (Architect i2000, Roche, Mannheim, Germany) and eosinophils count were measured in the blood (Beckman coulter LH750, USA). AERD subjects were given dexamethasone before surgery. Patients who volunteered for nasal lavage collection were asked to discontinue the use of both oral and nasal corticosteroids for seven days. However, to prevent the development of asthma symptoms, bronchodilators and mouth-inhaled aerosol corticosteroids were not withheld. The study was approved by the Bioethics and Science Committee in Research, with protocol number B02-14 and the Institutional Review Board at the National Institute of Respiratory Diseases (INER) Ismael Cosio Villegas.

### 2.3. Cell Culture

Respiratory tract epithelial cells were isolated from nasal mucosa (nasal polyps), which were obtained from AERD patients undergoing surgery for therapeutic reasons. Control cells were isolated from healthy tissue from the same group of patients with rhinosinusitis and subjects undergoing surgery for non-clinical reasons. Briefly, the nasal polyps were dissected to isolate the epithelial layer and were washed with saline to remove blood and other impurities; tissues were placed in 10mL DMEM and subsequently gently centrifuged at 3200× *g* for 10 min at 37 °C. The tissue was macerated with scissors and forceps, and 0.25% trypsin EDTA was added to dissolve mucus. Subsequently, tissue samples were incubated at 37 °C for 60 min. Tissue particles were then sieved using a cell strainer to isolate the cells. Next, the filtrate was centrifuged at 4300× *g* for 10 min. The pellet-containing epithelial cells were resuspended into culture flasks containing complete medium (Bronchial Epithelial Cell Growth Medium Bullet Kit (BEGM), Clonetics, CA, USA) with antibiotics (Penicillin-Streptomycin 100 µg/mL, Nystatin 100 U/mL; Invitrogen Life Technologies, Gaithersburg, MD, USA)and were then incubated at 37 °C, at 5% CO_2_. When NAEC reached 80% confluence, they were harvested using trypsin EDTA and incubated at 37 °C incubator for 10 to 15 min and observed under microscope; gentle tapping on the sides loosened the cells. BEGM with serum was added to arrest trypsin EDTA activity and was centrifuged at 3200× *g* for 10 min. The pellet-containing epithelial cells were dissolved with a small volume of 0.01 M PBS, and 2–3 volumes of RNAlater (Invitrogen Life Technologies, Gaithersburg, MD, USA) were added and stored at −20 °C until RNA extraction.

### 2.4. RNA Extraction and Quantification

Samples were thawed and centrifuged to remove RNAlater. Cells were then lysed and homogenised. RNA extraction was performed using the Qiagen RNeasy micro kit using the standard protocol on Qiacube (Qiagen, Mexico City, Mexico). RNA and cDNA quantifications were done using Qubit 3.0 fluorometer (Invitrogen Life Technologies, Gaithersburg, MD, USA) using the manufacturer’s instructions. Concerning quality assurance, the RNA Integrity Number (RIN) of RNA obtained from patient samples was determined using microfluidics analysis on the Agilent bio-analyser Pico RNA and HS DNA kits. Only samples with a RIN greater than 7 were subjected to RNA-seq. RNA quantification was performed at Translational Oncology, University Medical Center of the Johannes Gutenberg University, Mainz.

### 2.5. RNA Sequencing and Data Analysis

Library preparation was performed using the Illumina Truseq RNA V2 kit according to the manufacturer’s protocol. Libraries were clustered using TruSeq PE Cluster Kit v3-cBot-HS, and RNA-Seq was performed on Illumina’s HiSeq2500, generating paired-end 2 × 50 nucleotide reads using TruSeq SBS Kit v3 – HS. Basecalling and demultiplexing was performed with Illumina bcl2fastq v2. The read mapping to specific regions of the reference genome (GRCh38) was done with hisat2-2.0.1beta. A flexible overlap approach was used for *read counting and RPKM calculation with in-house software*. Upregulated genes should have a logarithmic fold change > log2 (1.5) and downregulated genes < −log2 (1.5). The *p*-values were adjusted stepwise using the Benjamini–Hochberg procedure. All genes passing an adjusted p-value cut-off of 0.05 are displayed in a heatmap. Pathway enrichment analysis was done using the 364 genes as input for the R package generally applicable gene set enrichment (GAGE). DESeq2 was used for differential expression analysis. RNA sequencing was carried out at Translational Oncology, University Medical Center of the Johannes Gutenberg University, Mainz. Submission of RNA-Seq data was achieved as per the instructions on the NCBI GEO webpage. Briefly, a metadata spreadsheet was filled with descriptions of samples and procedures. The MD5 hash checksum Mac OS app was used to create MD5 checksums to check the integrity of the raw data files of each sample. The metadata spreadsheet, compressed raw data and processed data files of each sample and supplementary data were uploaded to the NCBI GEO repository using the Filezilla FTP server. Files were processed by GEO curators and a unique accession number GSE158277 was issued by email within 5 days of submission.

### 2.6. Immunohistochemistry

Nasal polyp biopsies were fixed in 10% paraformaldehyde for 18 h. After fixation, the tissue block was embedded in paraffin, then cut in a microtome to 0.4 μm sections and affixed on AEPS (amino-propyl-tri-ethoxy-silane)-coated glass slides. Then, the slides were deparaffinised, rehydrated by washing with xylene and 50% to 100% ethanol concentrations, and finally rinsed with running cold tap water. Heat-based antigen retrieval was done with tris EDTA buffer. Blocking non-specific binding was achieved by incubating the slides with 10% normal serum, and immunohistochemical staining was performed using the Vectastain ABC kit (Vector laboratories) according to manufacturer’s instructions. Sections were incubated with primary antibodies against DMRT3 (PA5-85040; Thermo Fisher, South San Francisco, CA, USA in 1:500 dilution for 40 min. Subsequently, the slides were incubated with biotinylated secondary antibody anti-mouse/rabbit IgG for 30 min to detect DMRT3 in the biopsies. The sections were counterstained using Mayer’s haematoxylin (Vector Laboratories Inc., Burlingame, CA, USA) and DPX-mounted before observation under a microscope.

### 2.7. Nasal Lavage

Nasal lavages were performed by instilling 10mL of sterile physiological saline solution into each nostril; the fluid was expelled after 10s. About 8 mL of the nasal secretion was collected routinely from each patient (the extent of polyps did not modify the procedure or the collected volume). Nasal lavage samples were homogenised by vigorous shaking and centrifuged at 17,300× *g* for 10 min, then maintained at 20 °C until their use. Before ELISA measurements, nasal lavages were concentrated 8 times using Amicon^®^ Ultra centrifugal filter (Millipore, Billerica, MA, USA). The concentrating procedure was performed according to the manufacturer’s protocol.

### 2.8. DMRT3 ELISA Measurements

Measurements of DMRT3 were performed in 8 times concentrated nasal lavage fluid using a two-antibody sandwich ELISA (MyBioSource, San Diego, CA, USA) as previously described [12,13]. The concentration of DMRT3 in samples was calculated from the standard curve. The sensitivity of this kit is 10pg/mL. The detection range of this kit is 62.5 pg/mL–2000 pg/mL.

### 2.9. Statistical Analysis

Descriptive statistics were expressed as median (interquartile range); the nonparametric Mann–Whitney U test was used for comparison of concentrations between the groups. Graphs were calculated using STATA statistical software, version 13 (Stata Corp LP, College Station, TX, USA), *p* < 0.05 was considered statistically significant.

## 3. Results

### 3.1. Characteristics of Participants

In this study, we investigated the transcriptome of nasal epithelial cells derived from 12 patients (group A) suffering from AERD nasal polyps (NNPEC) compared with nasal epithelial cells from the non-affected nasal area from the same patients (NNAEC) in order to reduce genetic heterogeneity (AERD patients themselves served as a control). Nasal epithelial cells were successfully isolated from all subjects except for three control nasal mucosal tissues (nasal tissue was inadequate for cell culture from two subjects, and in one patient, the disease was severe so nasal mucosa was not collected). All patients were given 8 mg dexamethasone before the nasal polypectomy to prevent bleeding during surgery. Two new separate groups of subjects participated in the study for validation experiments, including the second group of AERD patients suffering nasal polyps (*n* = 12) and a healthy control group (*n* = 8) who underwent nasal lavage. All group B and C subjects were neither given dexamethasone nor were taking oral steroids. Furthermore, AERD patients stopped inhaled steroids 7 days before the nasal lavage to ensure corticosteroids did not affect mediator measurements. Clinical characteristics are shown below (Table 1).

### 3.2. Differential Gene Expression Analysis

**Figure 1:**
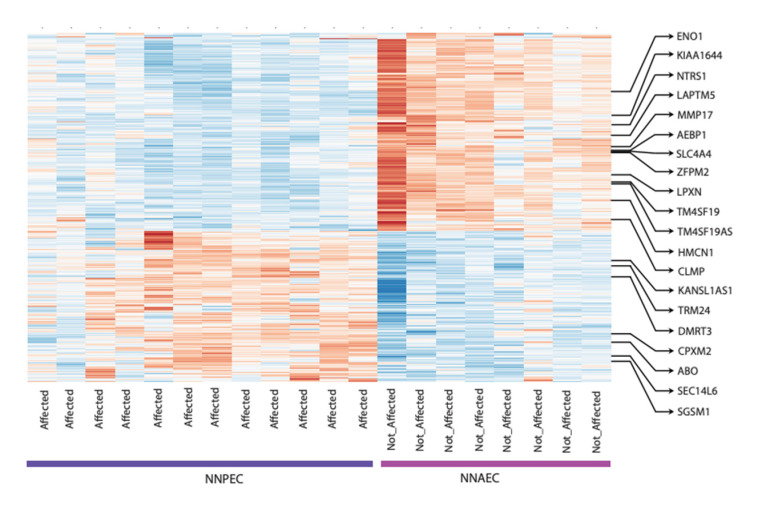
RNA-Seq results are clustered in this heatmap, showing differential expressions of AERD nasal polyp epithelial cells (NNPEC) versus AERD non-affected nasal area epithelial cells (NNAEC). In GEO, these two groups are annotated as affected vs not affected.

Airway epithelial cells derived from both AERD polyp and NNAEC mucosa were successfully sequenced. RNA sequencing of NNPEC versus NNAEC differentially expressed a total of 364 genes. The shown data represents the regularised logarithm (rlog) transformed count data. For plotting, the rows are scaled to have a mean of zero and standard deviation of one (z-score). (Figure 1). However, only 20 genes achieved statistical significance after Benjamini–Hochberg adjustment for false discovery rate: 8 genes were upregulated, and 12 genes were downregulated (Table 2). Functional analysis of these genes using GO analysis revealed categories that are associated with retinol metabolism, steroid hormone biosynthesis, primary bile acid biosynthesis, phenylalanine metabolism (Table 3), plasma membrane organisation, macroautophagy, leukotriene metabolic process, protein localisation to the membrane, drug catabolic processes, and neuron cell-cell adhesion. In addition, the Pathview toolset (Bioconductor) was used for pathway-based data integration and visualisation (Table 4). The data discussed in this publication have been deposited in NCBI’s Gene Expression Omnibus [14] and are accessible through GEO Series accession number GSE158277. Of particular interest was the identification of DMRT3.

### 3.3. Measurements of DMRT3 

**Figure 2:**
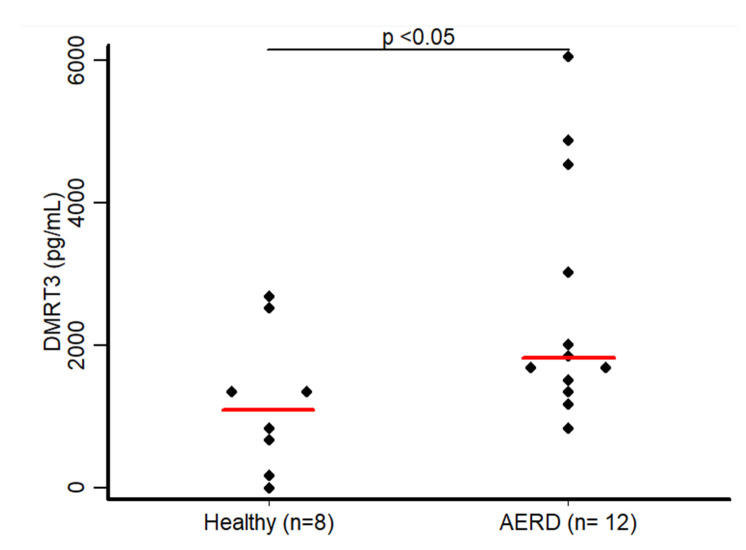
ELISA results are represented in this dot plot showing a significant difference in DMRT3 levels in nasal aspirates of AERD vs normal controls. Horizontal lines represent median values.

In order to investigate whether DMRT3 is released into the airway epithelial lining fluid, we performed ELISA measurements in the nasal lavages of patients suffering from AERD. A group of normal healthy subjects participated as control. Interestingly, AERD patients’ nasal lavage samples showed significantly elevated DMRT3 levels compared with normal controls (457 pg/mL (363–942 pg/mL) versus 275 pg/mL (275–610 pg/mL). (Figure 2). This finding is in agreement with the high differential gene expression observed in the RNA-Seq experiment.

### 3.4. DMRT3 Immunoreactivity in Nasal Polyps

**Figure 3:**
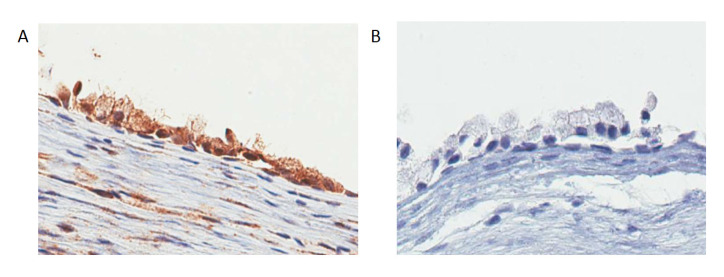
(**A**) Immunohistochemistry predominantly localises DMRT3 in airway epithelia of nasal polyps from AERD patients. (**B**) Negative control for DMRT3. A representative experiment of one of three nasal polyps.

To investigate DMRT3 immunoreactivity in nasal polyps, we applied immunohistochemistry to nasal polyp biopsies derived from AERD patients. DMRT3 immunoreactivity was localised predominantly to the airway epithelium (Figure 3) and there was sparse immunoreactivity localised to mononuclear cells in the subepithelial tissue.

## 4. Discussion

Nasal polyp management remains a significant challenge in AERD. In the present study, we investigated the transcriptome of nasal airway epithelial cells and identified several differentially regulated genes associated with retinol metabolism, steroid hormone biosynthesis, primary bile acid biosynthesis, and phenylalanine metabolism. Of particular interest was the identification of DMRT3, which is a gene involved in embryonic development. The DMRT gene family, a class of molecules, is characterised by a signature zinc finger-like DNA-binding motif known as the DM domain. Interestingly, by ELISA, we demonstrated that DMRT3 is released in increased concentration in the upper airways of AERD patients, suggesting it is involved in the pathogenesis of this disease. To our knowledge, this is the first study to utilise RNA-Seq gene expression profiling of AERD polyp-derived epithelial cells.

Medical treatment of nasal polyps in AERD patients includes topical and oral corticosteroids, antibiotics and surgical intervention. However, up to 40% of NP patients show recurrence and require an additional endoscopic sinus surgery within 18 months [15]. Clinical trials with novel therapeutic biologics have recently tested anti-IL-5, anti-IL-4, anti-IL-13 and anti-IgE. However, most of these treatments have a limited impact on nasal polyps [16,17,18]. Peng et al. showed defective host defences and heightened inflammation responses in whole tissue nasal polyps using RNA sequencing [19].

To investigate local transcriptomic changes implicated in nasal polyp pathogenesis, we performed sequencing of nasal polyps and healthy nasal epithelial cells from AERD patients that revealed 20 differentially regulated genes. Of particular interest the DMRT3 gene, which was significantly over-expressed in NNPECs. This gene is a member of the DMRT family, known to play a conserved role in sex determination, sexual dimorphism and other aspects of sexual reproduction [20,21,22]. In addition, we investigated whether DMRT3 is released into airway epithelial lining fluid; we have measured this protein concentration in nasal fluid. The epithelial lining fluid (ELF) forms a thin fluid layer covering the nasal mucosa and reflects the ongoing changes in some of the pathological processes related to the progression of disorders in the upper airways. In this study, measurements were performed in the second group of AERD patients and compared with normal subjects. In contrast to AERD patients who underwent nasal polyp removal, this new group of AERD subjects discontinued the use of oral steroids, excluding the possibility of gene expression alteration due to steroids. Interestingly, DMRT3 levels were increased in the nasal lavages of AERD patients compared with healthy subjects, which infers this protein is released in the upper airways. Moreover, we further demonstrated that DMRT3 immunoreactivity is predominantly localised to the nasal epithelium by immunohistochemistry, further supporting its synthesis by this cell type. DMRT3 is localised in nucleus; however, this protein was detected in both the nucleus and the cytoplasm of airway epithelial cells. Consistent with our findings, Tsai et al. has shown DMRT3 immunoreactivity to both the nucleus and cytosol of testis cells [23]. As we also detected increased DMRT3 immunoreactivity in the nasal fluid, this molecule may have been released from the nucleus to the extracellular space. To our knowledge, this is the first report showing DMRT3 is produced in the upper airways in humans.

We hypothesise that DMRT3 may be involved in the genesis of nasal polyp formation. Evidence for the role of DMRT in embryogenesis derives from a zebrafish embryo model showing a restricted expression pattern of DMRT3 in the neural tube, olfactory placode and germ cells of both undifferentiated and adult gonads [24]. In humans, DMRT1, DMRT3 and DMRT2 (9p24.3) have been associated with human testicular dysgenesis and XY male-to-female sex reversal [25,26]. In vitro studies have shown that DMRT3 serves as a transcription factor to control estrogen receptor 1 (ESR1) promoter activity in the nucleus: it promotes the binding of estrogen receptor 1 (ESR1) mRNA to their 2ʹ, 5ʹ-oligoadenylate synthetase 3. Interestingly, mutation of DMRT3 induces higher ESR1 promoter activity than wild-type DMRT3 [23]. Similarly, DMRT3 has been associated with lung squamous cell carcinoma (LUSC). To unravel the mechanisms of DMRT3 in this disease, Zhang et al. developed a one-class support vector machine (OC-SVM) to predict transcription factor (TF) targets [27]. Interestingly, they found high DMRT3 expression restricted mainly to samples over-expressing the transcription factors SOX2 and TP63, with both SOX2 and DMRT3 targeting the TP63 promoter. The authors proposed that DMRT3 may participate in the TP63/SOX2 circuit for regulating squamous cell differentiation and survival, and that these three factors may co-regulate gene functioning in human LUSC development. Over-expression of SOX2 has been reported in nasal polyp epithelial cells [28]. SOX2 is one of the transcription factors which is essential for the pluripotent cell development and maintenance of undifferentiated embryonic stem cells [29]. Our study is the first to demonstrate an significant over-expression of DMRT3 associated with nasal polyps in AERD patients. Thus, DMRT3 along with SOX2 may regulate nasal polyp differentiation. To date, however, the role of TP63 in nasal polyps remains to be shown. Interestingly, surgical removal of nasal polyps improves asthma symptoms and decreases urinary LTE_4_ levels [8,30]. Thus, the development of new drugs that neutralise this gene may antagonise nasal polyp formation and reduce asthma symptoms.

Several additional genes were differentially regulated in the current study, and the number of genes downregulated was higher than upregulated in AERD polyps, hinting at dedifferentiation and trans differentiation [31] phenomena. For example, NTSR1 inhibition induces intrinsic apoptosis via downregulation of Bcl-w and Bcl-2 in glioblastoma cells [32]. Neurotensin is the ligand to NTSR1. Kontovounisios et al. showed lower levels of this mediator in adenomatous polyps than in adenocarcinoma [33,34]. On the other hand, Brun et al. demonstrated that neurotensin significantly increased COX-2 mRNA levels by 2.4-fold and stimulated PGE_2_ release in HT-29 cells [35]. Thus, neurotensin and NTR1 are part of the network activated after mucosal injuries, and NT stimulates epithelial restitution at least, in part, through a COX-2 dependent pathway. Interestingly, NTSR1 was significantly downregulated in AERD polyps compared to non-polyp mucosa, and the retinoic acid metabolism pathway was significantly enriched in our results, suggesting that downregulation of NTSR1 might influence retinol metabolism in AERD polyps [36]. Retinoids are essential for the maintenance of epithelial differentiation. Retinoic acid was first implicated as a signalling molecule based on its teratogenic effects on limb patterning [37]. As such, retinoids play a fundamental role in the chemoprevention of epithelial carcinogenesis and differentiation therapy. Degradation of retinol in AERD polyps suggests a dedifferentiation phenomenon.

Adipocyte enhancer-binding protein 1 (AEBP1) is a multifunctional protein involved in regulating critical biological processes, including adipogenesis, mammary gland development, inflammation, cholesterol homeostasis, atherogenesis and cancer [38]. Xing et al. showed that AEBP1 is overexpressed in COAD tissues and cells and that the expression of AEBP1 was correlated with tumour size, the level of histologic differentiation, lymph node metastasis, and cancer stage in COAD patients [39]. AEBP1 also promotes the epithelial-mesenchymal transition of gastric cancer cells by activating the NF-kB pathway; this, in turn, predicts poor patient outcomes [40]. AEBP1 overexpression results in uncontrollable activation of NF-κB, which may have severe pathogenic outcomes. In macrophages, NF-κB activation leads to the release of various proinflammatory mediators [41]. AEBP1 also functions as a transcriptional repressor of the adipose P2 (aP2) gene in preadipocytes, and its expression is downregulated during adipocyte differentiation [38,42]. This molecule binds to the adipocyte enhancer-1 (AE-1) DNA sequence on the aP2 gene, leading to the transcriptional repression of aP2. Interestingly, aP2 is also expressed in macrophages, and it has a significant role in their biological responses. Studies illustrates the ability of AEBP1 to induce transcriptional regulation of macrophage cholesterol homeostasis [43] by suppressing the activity of PPARc1 and LXRα in these cells. AEBP1 downregulates the activity of PPARc1 and LXRα by binding to the AE-1 homologous sequence. Both macrophage infiltration and NF-kB activation play an important role in nasal polyps [44,45]. However, the role of AEBP1 in AERD nasal function remains to be shown.

Other down-regulated genes include LPXN, ENO-1, TM4SF19, CLMP, SLC4A4, ZFPM2, and HMCN1. Leupaxin (LPXN) is vital in tumourigenesis and the progression of bladder cancer [46] by upregulating the expression of S100P via the PI3K/AKT pathway. LPXN also plays a role in regulating hepatocellular carcinoma progression, at least in part, by enhancing beta-catenin transcription activity [47]. Similarly, enolase 1 (ENO1), a glycolytic enzyme, is found to be upregulated in gastric cancer (GC) cells and is associated with poor prognosis in GC patients [48]. To evaluate the effect of ENO1 on the glycolysis pathway in GC cells, Yang et al. analysed the glycolysis changes caused by ENO1 [49]. They found that overexpression of ENO1 significantly enhanced cells’ capability for glycolysis. In contrast, silencing of ENO1 decreased the glycolysis capacity of GC cells. In breast cancer cells, inhibition of alpha-enolase affects the biological activity of breast cancer cells by attenuating the PI3K/Akt signalling pathway [50]. TM4SF1, also named OCTM4, belongs to the transmembrane 4L six superfamily and is associated with gastric carcinoma. Indeed, down-regulation of TM4SF is associated with the metastatic potential of gastric carcinoma [51,52]. In contrast, it is known that TM4SF can interact with integrin to affect various cellular functions and mediate signal transduction events that are associated with numerous physiological functions, such as cell development, activation, growth and motility [53]. The mechanisms by which TM4SF regulates in GC metastasis remains to be shown. CLMP, also known as the coxsackievirus and adenovirus receptor (CAR), serves as a viral receptor and is a component of tight junctions; it plays an important role in tissue homeostasis [54]. Mutations of CLMP are associated with congenital short-bowel syndrome [55]. In relation to SLC4A4, variants of this gene may cause impairment of bicarbonate reabsorption in the proximal renal tubules, causing a decrease in the threshold of renal bicarbonate and resulting in proximal renal tubular acidosis [54]. SLC4A4 is a sodium bicarbonate co-transporter that mediates the coupled movement of sodium and bicarbonate ions across many cells’ plasma membrane, including airway epithelial cells [56]. The known biological changes implicated in nasal polyp formation in AERD patients involves many processes, including airway inflammation, tight junction impairment, pathogen infection, signalling and defective host defence mechanisms. However, future studies should further decipher the role of these genes in AERD.

The ZFPM2 protein, also named Fog2, was identified as a biomarker of malignant pleural mesothelioma and has been associated with ovarian tumours and neuroblastoma [57,58]. Interestingly reduced ZFPM2 (Fog2) expression has been reported in the glomeruli of diabetic mice as well as in TGF-β-treated mouse mesangial cells (MMC) [59]. In this study, FOG2 knockdown by siRNAs in MMC activated Akt and increased the protein content/cell ratio. Thus, downregulation of FOG2 under diabetic conditions can augment Akt kinase activation and subsequently result in glomerular mesangial cell hypertrophy. Similarly, AKT is expressed strongly in nasal polyps [60]. Moreover, the composition of the ECM in nasal polyps is different from that of normal nasal tissue [61]. Furthermore, this process of altered tissue remodelling in NP has direct effects on the biomechanical properties of the polypoid tissue, facilitating tissue growth and oedema. In this regard, hemicentin (HMCN), which was also downregulated, is a member of the fibulin family of extracellular matrix proteins [62]. Thus, the expression pattern of ZFPM2 and HMCN provides the preliminary view, and the exact role of the investigated genes in AERD nasal polyp epithelial cells requires further investigation.

To date, this is the first study to analyse the transcriptome of airway epithelial cells derived from nasal polyp and non-nasal polyp tissue derived from the same patients. Strengths of having analysed matched samples include (a) isolating NNAECs from the same subjects provides reasonable control for individual differences and, as a result, prevents random error. Most previous studies have used nasal tissue. For example, Stevens et al. present a general analysis of gene expression levels using scRNA-seq comparing NP of AERD and CRSwNP patients. ALOX15 was predominantly expressed by apical epithelial cells and significantly increased in AERD compared to CRSwNP [62]. They reported that downstream mediators in the 15-LO pathway, including 15-HETE and 15-oxo- ETE, may be important factors contributing to AERD pathophysiology. They also discovered a trans-metabolic process in which epithelial cells convert AA to 15-HETE (by 15-LO), and nearby mast cells convert 15-HETE to 15-oxo-ETE (by HPGD); (b) genetic variability is reduced; (c) a large sample size is not required as the sampling is done repeatedly from the nasal passage of the same individual. Future studies should investigate the role of DMRT3 in other nasal polyp-related diseases to define whether this gene is specific to AERD or if it may be involved in nasal polyps of other origins such as cystic fibrosis and allergy, among others. Our study has some potential limitations. For example, the control group (group C) was younger than the AERD groups, i.e., group A and B. However, both groups B and C were used for the validation studies showing increased levels of DMRT3 in nasal lavage. This finding demonstrates that DMRT3 is expressed in NP tissue and released into nasal airway lining fluid.

In summary, the present study has revealed 20 differentially expressed genes in NNPEC using RNA sequencing. Of particular interest was the identification of DMRT3, which is a gene involved in embryonic development. ELISA measurements showed DMRT3 is released in high concentrations in the nasal airway epithelial fluid of AERD patients. This study further confirms the value of performing a matched-case control approach as a valuable tool to uncover the mechanism involved in AERD polyps, and it demonstrates that careful study design allows for the uncovering of local gene activation. Overall results suggest that DMRT3 may become a potential molecular target for therapeutic intervention in AERD.

## Figures and Tables

**Table 1:** Clinical characteristics of subjects.

Samples Analysed	Nasal Polyp Epithelial Cells (RNA Sequencing)	Nasal Lavage (Validation by ELISA)	
Group of Patients	Group A * AERD	Group B AERD	Group C (Healthy Controls)
No. of patients	12	12	8
Sex female/male	9/3	9/3	5/3
Age (years), median (range)	54 (41–55)	43 (33–51)	29 (26–39)
FEV1 (% predicted), median (range)	87 (86–89)	94 (90–97)	97 (84–109)
Eosinophils (cells/mm^3^), median (range)	250 (150–600)	300 (300–400)	175 (90–200)
IgE (UI/L) median (range)	67.3 (57–285)	125 (90–195)	26 (9–106)
Systemic steroids	Dexamethasone 8 mg single dose	None	N.A.
High Grade of Nasal Polyps * *n* (%)	12 (100%)	7 (58%)	N.A.
Severe Asthma ** *n* (%)	8 (66%)	10 (83%)	N.A.

* Nasal epithelial cells from the non-affected nasal area (NNAEC)from group A were also isolated (AERD patients themselves served as control in the RNA sequencing experiments). ** Asthma that requires high medication to get control. N.A., not applicable.

**Table 2:** Differentially expressed genes with log 2-fold change.

Serial No.	Rownames(resOrdered) [1:20]	Log 2-Fold Change	*p* Value Adjusted
1	Adipocyte enhancer-binding protein 1 AEBP1	−2.7073627	2.028219 × 10^−6^
2	Matrix metalloproteinase-17 MMP17	1.7718316	2.076115 × 10^−6^
3	Carboxypeptidase X CPXM2	2.4652531	2.076115 × 10^−6^
4	Coxsackie- and adenovirus receptor-like membrane protein CLMP	−1.9817694	2.076115 × 10^−6^
5	Neurotensin receptor 1 NTRS1	−2.4125046	3.685087 × 10^−5^
6	Doublesexand mab-3 related transcription factor 3 DMRT3	2.3528259	5.251815 × 10^−5^
7	Transmembrane 4 L six family member 19-antisense RNA 1 TM4SF19-AS1	−2.1926320	6.310982 × 10^−5^
8	Alpha 1-3- N -acetylgalactosaminyltransferase and alpha 1-3-galactosyltransferase ABO	1.5809817	6.733651 × 10^−5^
9	Shisa-like 1 KIAA1644	−2.3470082	9.386238 × 10^−5^
10	Hemicentin 1 HMCN1	−2.2726469	1.158004 × 10^−4^
11	Lysosomal protein transmembrane 5 LAPTM5	−2.2271424	1.616751 × 10^−4^
12	Zinc finger protein, FOG family 2 ZFPM2	−2.2228298	3.081244 × 10^−4^
13	Small G protein signaling modulator 1 SGSM1	2.0366465	6.555131 × 10^−4^
14	Leupaxin LPXN	−1.9954138	9.258335 × 10^−4^
15	Enolase 1 ENO1	−0.8026367	9.774806 × 10^−4^
16	Transmembrane 4 L six family 19 TM4SF19	−2.0284742	1.174304 × 10^−3^
17	Solute carrier family 4 member 4 SLC4A4	−2.0260924	1.578034 × 10^−3^
18	Tripartite motif containing 24 TRIM24	0.6134868	1.578034 × 10^−3^
19	KAT8 regulatory NSL complex subunit 1-antisense RNA 1 KANSL1-AS1	1.6215603	2.162777 × 10^−3^
20	SEC14-like lipid binding 6 SEC14L6	1.9542750	2.175972 × 10^−3^

**Table 3:** Pathways enriched between NPEC versus NNAE using pathview.

Serial No.	ID	Pathway	*p* Value
1	hsa00830	Retinol metabolism	0.006416688
2	hsa00140	Steroid hormone biosynthesis	0.017377887
3	hsa00120	Primary bile acid biosynthesis	0.017620697
4	hsa00360	Phenylalanine metabolism	0.04440799

**Table 4:** Pathways enriched between NPEC versus NNAE using GO enrichment analysis.

Serial No.	ID	Pathway	*p* Value
1	GO:0007009	Plasma membrane organisation	0.006603899
2	GO:0016236	Macroautophagy	0.009654063
3	GO:0006691	Leukotriene metabolic process	0.011124361
4	GO:0072659	Protein localisation to plasma membrane	0.012383519
5	GO:0042737	Drug catabolic process	0.015426792
6	GO:0007158	Neuron cell-cell adhesion	0.017589775
7	GO:0009812	Flavonoid metabolic process	0.022826975
8	GO:0000045	Autophagic vacuole assembly	0.023120577
9	GO:0048713	Regulation of oligodendrocyte differentiation	0.023746775
10	GO:0000042	Protein targeting to Golgi	0.024045644
11	GO:0000301	Retrograde transport, vesicle recycling within Golgi	0.024312565

## Data Availability

The data presented in this study are openly available in the NCBI GEO repository (accession number GSE158277).

## Abbreviations

AERD	aspirin-exacerbated respiratory disease
Bcl	B cell lymphoma 2
BEGM	bronchial epithelial cell growth medium
cDNA	complementary DNA
CNS	central nervous system
COX	cyclooxygenase
CRSwNP	chronic rhinosinusitis with nasal polyps
CysLT	cysteinyl leukotrienes
CysLTR1	cysteinyl leukotriene receptor 1
DMEM	Dulbecco’s modified Eagle’s medium
DNA	deoxyribonucleic acid
EC	epithelial cell
ECM	extracellular matrix
ELF	epithelial lining fluid
ELISA	enzyme-linked immune sorbent assay
EMT	epithelial-mesenchymal transition
ESCC	esophageal squamous cell carcinoma
FEV1	forced expiratory volume in 1 s
GEO	Gene Expression Omnibus
GO	Gene Ontology
GRCh38	Genome Reference Consortium Human Build 38
HETE-5	hydroxyeicosatetraenoic acid
HPGD-15	hydroxyprostaglandin dehydrogenase
HT	high-throughput
Ig E	immunoglobulin E
IHC	immunohistochemistry
LTE4	leukotriene E4
NAEC	nasal airway epithelial cells
NCBI	National Center for Biotechnology Information
N-ERD	NSAID-exacerbated respiratory disease
NNAEC	non-affected nasal area epithelial cell
NNPEC-N-ERD	nasal polyp epithelial cell
NP	nasal polyps
NPEC	nasal polyp epithelial cell
NSAID	non-steroidal anti-inflammatory drug
NSCLC	non-small cell lung cancer
Oxo-ETE	oxoicosatetraenoic acid
PBS	phosphate-buffered saline
PGE2	prostaglandin E2
RIN	RNA integrity number
